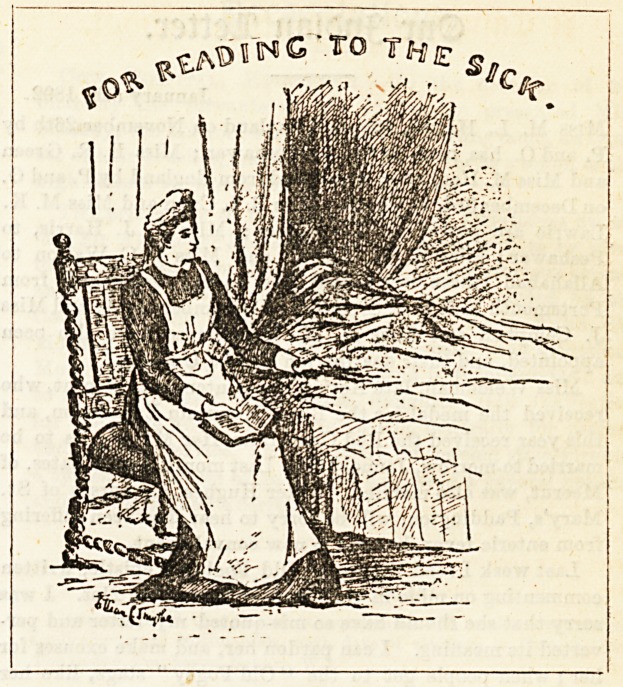# The Hospital Nursing Supplement

**Published:** 1892-02-06

**Authors:** 


### "The Hospital" Nursing Mirror.

Being the Extra Nursing Supplement of "The Hospital" Newspaper.

Contributions for this Supplement should be addressed to the Editor, The Hospital, 140, Strand, London, W.C., and should have the word "Nursing" plainly written in left-hand top corner of the envelope.

### En Passant.

**Perth Nursing Society.**—At the annual meeting held last month, the Chairman announced that they hoped to affiliate with the Jubilee Institute in the course of the year. The expenditure for 1891 had been £209. The amount of work done during the year was as follows: old cases on register on January 1st, 1891, 36; new cases, 298—total, 334. Visits made, 6,064. Patients cured, 182; relieved, 62; died, 41; sent to Infirmary, 10; sent to Hill-side Home, 3; on roll, December 31st, 1891, 36—334. Sister Bowley and Nurse Williamson have worked well.

**Buchanan Cottage Hospital.**—The Matronship of this hospital, at St. Leonards-on-Sea, will be vacant in April, as the Matron, Miss Burwood, is about to be married. The Buchanan was founded in 1881, and has advanced steadily ever since. With 16 beds in the general wards, and three beds in private wards, the hospital dealt with 194 in-patients last year, and over 100 operations were performed. In addition, an out-patients' department was opened last year, and this has added considerably to the work of the hospital. The Matronship, which has been most ably filled, is by no means a sinecure, as there is no resident house surgeon.

**A Lesson for the Public.**—The influenza is decreasing, and where 70 applications for nurses were received ten days ago, only 50 are now received. But the public has got a fright and we will also hope a lesson; the demand for nurses has never before been so great nor so persistent, for the medical men were the first to say when called in—"Much depends on the nursing." That there are not enough trained nurses in the United Kingdom all doctors know, that 500 women apply for each vacancy for training in a good hospital all matrons know; what the public has to learn is that they must for their own sakes, if for no less selfish reason, help forward the training of nurses. We have always strongly upheld that funds given to a hospital are given primarily to the patients, and that to use these funds for supplying aught but absolute necessities for the nurses was wrong. But why should not some of the rich and generous of the land give their money directly towards the building of homes in connection with hospitals where the nurses could be trained? Here are hundreds of women who are anxious to earn their living as nurses rather than as clerks or typewriters where they would have to compete with men, yet they are unable to get the training they desire. Newcastle has 270 beds in its Infirmary, but, for reasons best known to its Governors, it does not train probationers. Workhouse infirmaries containing hundreds of beds and the most cruelly limited attendance on the sick paupers who occupy them, do not train probationers. Here are the women, here are the cases from which they could learn, rich and poor would alike benefit by the learning and the lesson taught. Let the public then think seriously whether money given towards training homes for nurses would not be worthily bestowed. The doctors would certainly think so, for the half-trained or wholly untrained women whom in the present dearth some of them have employed, have proved when real danger arose, not only incapable but actually mischievous. In fact, an incompetent nurse is worse than no nurse, and if in future epidemics the rich desire skilled attendance, they had better use their influence and their wealth towards more homes for nurses in connection with hospitals.

**Short Items.**—Miss Florence Nightingale has returned to town. Though always more or less of an invalid, she is now enjoying fair health.—It cost the guardians of Kingston half a-year's salary in advertising to procure a nurse, and in the end they appointed Miss I. C. Cole (untrained).—A cottage at Galashiels has been given as a Home for the Queen's nurses.—We this week chronicle a presentation which is another mark of the advance of the asylum attendant. The London County Council has during its existence granted to all attendants a fortnight's holiday, and slightly increased their wages. Now the County Council should insist on lectures being delivered to the attendants, and books of instruction supplied.

**Sheffield Workhouse Hospital.**—On Monday evening last the nurses of the above institution gave their annual entertainment to the inmates, which consisted of a tuneful cantata, entitled, "Flora's Festival." There was a large assembly of Guardians, visitors, and officers. The cantata occupied the best part of two hours. From the commencement all went smoothly, and the audience testified their high appreciation by frequent outbursts of applause. Nurse Lightowler was an admirable Queen Flora, and sung the music allotted to her with rare taste. The other characters were effectively represented, while the choruses went forward with a commendable smoothness. From a spectacular point of view, the performance had many pleasing features, for Mr. Stokes had borrowed some scenery from the Alexandra Theatre. Nurses Chawner, Brown, Gardner, and Pulham sung their parts and solos with effect; while Nurse Dinsdale ably discharged the duties of accompanist. The officers and nurses afterwards had a dance, and thus brought a very pleasant evening to a close.

**Glasgow Nurses.**—The following extract from the report just issued by Glasgow Royal Infirmary Committee will be interesting to those who read about the late row: "In September last the attention of the managers was called to certain complaints by the nurses regarding their hours of work and diet. The managers, at a meeting held shortly before, had submitted to them a report by the Superintendent and late Matron recommending certain additions to the staff of nurses and cleaners with a view to the more efficient working of the hospital, the shortening the working hours of the nurses, and relieving them of all remaining menial duties. The managers were proceeding to carry out these recommendations, when the complaints referred to were brought under their notice, and, in order the better to do so, they resolved to make a thorough enquiry into the complaints, and appointed a special committee for that purpose. The committee held numerous meetings, examined a large number of nurses and officials, and presented a lengthened report, which was published at the time in the newspapers. The recommendations in the report were at once adopted, and with various other improvements relating to the nursing department have been or are in course of being carried out. The increase in the number of nurses involved the provision of additional sleeping accommodation and enlargement of the nurses' dining-hall. The managers regret to state that, owing to continual ill health, Miss Wood, who had acted as Matron for six years, was obliged to resign the matronship of the institution. They were fortunate, however, in securing the services of Mrs. Strong, who was formerly Matron for several years. Miss Alexander, the Superintendent of day nurses, who was also acting Matron during the illness of Miss Wood, resigned her office during the year, having been appointed Matron of the Paisley Infirmary; and Miss Love, formerly Superintendent of surgical night nurses, who had been appointed to succeed Miss Alexander, resigned her office recently for family reasons. The managers have pleasure in testifying to the very efficient way in which both these ladies discharged their duties. It is proposed to fill up the vacancy from the nursing staff."

### A Canadian Training School.

In the early history of the school the character of the instruction given seems to have been somewhat fragmentary, and therefore in order to obtain the necessary certificate of qualification at the expiration of two years, nurses were required to pass an oral examination only; but since the year 1885 there has been a regular course of study and lectures, lasting for nine months, in each year. At present nurses are required to pass an entrance examination at end of one month of trial, consisting of ordinary English and practical work. The English consists of reading, penmanship, and dictation, the practical work—how to make a hospital bed, bathe a patient in bed, change a slip, or draw sheet, take temperature, pulse, and respiration, prepare and apply a poultice, apply dry heat, prepare solutions of carbolic acid 1-20, and bichloride of mercury 1-1000. Those who give promise of ability are accepted, and after signing an agreement to remain two years, become pupils of the school, receive uniform, cap, and apron, and are called pupil nurses. Nurses who have spent one year in the various hospital wards, and have gone over the required course of study and lectures for the first year, are examined by the Superintendent of the school. This is a written examination. Those who are successful in obtaining not less than 50 per cent., if otherwise suitable, are placed in charge of either one or more wards. They then receive a black velvet band, which encircles the muslin cap, and are afterwards called head-nurses. At the expiration of the second year, if successful in obtaining 50 per cent. in each subject, at an examination before a medical board of examiners, nurses receive a certificate, signed by the examining board, the Medical Superintendent, and Lady Superintendent of the hospital; also a badge, bearing a beaver, the Canadian emblem, and the inscription, "Toronto General Hospital Training School for Nurses," and twenty-five dollars in money.

In 1887 a wing was added to the west end of the main building, which contains parlours, dining-room, and bedrooms. This is set apart expressly for the use of the nurses, and is commonly designated by them as "The Home." The parlours are bright and pretty, and contain a medical and general library, and besides this on the tables may be found a large number of monthly magazines and periodicals. In this "home" the nurses are entirely removed from hospital sights and sounds, during the period allowed for rest and relaxation, and on Wednesday evenings from 7.30 until 9.30 are allowed to receive their friends. The rising-bell rings at 6 a.m., and then each nurse is expected to rise, have her room in order, and be ready for breakfast at 6.30. At 7 a.m. they all assemble in the parlour for morning prayers. This service is conducted by the Superintendent, and consists of a hymn, a portion of Scripture, and a prayer, closing with the Lord's Prayer, in which all unite. During the day each nurse gets one hour off duty, besides meal-time. She also gets one afternoon off during the week, and one half of Sunday. Besides this she is allowed two weeks' holiday in each year. They are expected to be in their bedrooms at 10 p.m., and all lights extinguished at 10.30.

It is gratifying to those interested to know that the Toronto School has become a centre from which other hospitals have drawn. The number of those who now hold the certificate of this school is 131. Of these 29 hold positions in hospitals. The Kingston, London, Brockville, Belleville, Brantford, Collingword, Peterboro, and many other hospitals in Canada, as well as some in the United States, are superintended by nurses who were trained in this hospital. Many others are engaged in private nursing, and three are foreign missionaries in China, one in India, and one in Central Africa. The record of the school seems to be of branching influence in the world, and increased usefulness to the hospital from year to year, while the hospital offers ever increasing advantages in this educational work.

"Tis weary watching wave by wave,
And yet the tide heaves onward.
We climb like corals, grave by grave,
Yet pave a pathway sunward.
We're beaten back in many a fray,
Yet newer strength we borrow,
And when the vanguard rests to-day,
The rear shall camp to-morrow."

### On the Nursing of Children.

III.—EMERGENCIES.—(*Continued from page* ci.)

So the first thing to ensure is warmth; let the child be nursed in a shawl or blanket, and near the fire, by a person who must give her whole attention to soothing the poor little victim, getting him to swallow warm milk or any other hot drink which it will take.

In the meantime the nurse can rapidly get together such dressings as the house may contain. Oil is generally to be had, and is the best of all applications for the first dressing, and the doctor will decide what he wishes to succeed it. It is well not to remove the clothes until these preparations are made, and then the poor child can be attended to with the least possible delay, over what must be a painful business.

There should be foot-warmers and plenty of blankets, and a flannel nightgown if possible, and the sooner the child is quite warm the better. This is a point which needs constant repetition, for it is one that cannot be too strongly insisted upon. Valuable time immediately after an accident is often lost through lack of presence of mind in the bystanders. This applies equally to cases where children are run over, or have a bad fall. They should be *at once* placed in a recumbent position, the head not raised at all, but every care taken to get the feet and body warm, and to keep them so. The room should be perfectly quiet, and very little light should be admitted, and one person only should remain to watch for a return to consciousness. One person is (or should be) absolutely still, but two watchers are likely to talk, or, worse still, to whisper; therefore, for the child's sake, self-denial should be exercised by his anxious friends.

Nature does so much for children if she is left to do it; but when people interfere with her she resents it. To wait and do nothing is sometimes the wisest of all measures, and it is the hardest to practise.

Any child who falls on to his head or back, if from only a little height, should be watched and kept as quiet as possible; and if he shows the slightest sign of being the worse for his accident, no time should be lost in sending for a doctor. There is seldom sufficient attention paid to this class of injury when it first takes place. Another point needs mentioning with regard to accidents, for it is one constantly brought forward whenever one happens, more especially in the case of a child who has been run over. He is naturally dirty—covered with dust or mud, and the nurse's first instinct is to make him clean.

But this is not an "instinct" which must be followed. She must think first of warmth and absolute quiet, and last, of washing the little patient. That should never be attempted until she is quite sure that it is not only safe but desirable. It will do him no harm to remain in his bemired condition for awhile, but it may cause infinite mischief to the shaken brain and damaged little body if he is disturbed, however gently, for the process of cleansing.

Moreover, he may be dying, and what is more cruel, than to add to his last moments, one unnecessary discomfort. In all serious accidents this possibility is one that should weigh so heavily in our consideration of circumstances, that we should never, on any account, let our natural wish to make a patient "look comfortable," over-ride our knowledge that he would rather be "let alone" for the present.

Also a nurse must bear in mind that whilst endeavouring to obtain this absolute rest for the child whom she desires to help by all means in her power, to live, she must not lose sight of the fact that his individual tastes must be considered. If he returns to consciousness she will find him difficult to manage, but still it must be done! He must be coaxed and humoured, and kept without excitement, and if he desires to talk he may do so to please himself, but he must not be urged to speak, nor must he be questioned simply for the gratification of his companion's curiosity. The doctor will make any absolutely necessary enquiries when he comes, and that is quite soon enough for the little brain to be urged to remember anything.

For the doctor to arrive and find an injured child propped up, and being urged to swallow nourishment, must give him little confidence in the subsequent care bestowed on the patient. It will seem rather too late, then, to urge the recumbent position and the absolute quiet being insured!

All nurses are taught how to deal with hæmorrhage, but they do not always remember how very much alarmed a child is at the sight of even a little blood, and it is, therefore, desirable to prevent its seeing anything that can possibly be hidden from it of any wound.

Of course, it is also most undesirable to discuss such things in the presence of children, as they listen and understand a great deal more than is supposed.

In many ways a little child can be helped to bear his burden of pain, for his thoughts are easily diverted from remembrance of the sufferings recently passed through. He counts on sympathy, and when he feels assured of it his confidence in the nurse, who has never worried him needlessly, is almost boundless. If she considers his tastes and wishes in small things, he is generally quite willing to accept her verdict when she forbids what is harmful.

Children form views on all subjects with quite as much firmness as grown people, only, luckily for us, they are easier to guide into new paths, provided always that they are led, and not driven.

### County of Cornwall Trained Nurses' Home.

THE annual meeting of the subscribers to the Trained Nurses' Home and the Truro District Nurse Fund was, by kind permission of the Mayor, held at the Town Hall, Truro, on Thursday, January 28th. The chair was taken by the Lord Bishop at three o'clock, and after the minutes of the last meeting, and the report of the private nursing, and of the Truro district nursing for the year had been read, his Lordship expressed his pleasure at being present on the occasion, and his appreciation of the value of such work as was being carried on by this and similar institutions. He spoke of the blessing of skilled nursing both to rich and poor, of the gain to those who were able to offer themselves to the work as well as to those who were nursed by them, dwelling also upon the evidence afforded by such good works as this to the power of Christianity.

Canon Bourke, on behalf of himself and the clergy in the other parishes, spoke warmly of the district nursing done by Miss Rogers (the Superintendent) and the district nurse, and mentioned the warm expressions of gratitude from their patients. The election of President, Vice-president, Committee, &c., followed, and the meeting closed with the usual votes of thanks.

### IRRITABILITY.

How sadly a long illness tries our temper, and brings out irritability and other like faults and failings. When we were first taken ill we had the best intentions in the world not to give trouble, and to bear what came to us with equanimity and fortitude; but the constant recurrence of pain, and the ceaseless attentions our comfort required become so irritating that we at last break down altogether. To our surprise almost we find ourselves annoyed by trifles, the violent shutting of a door, or a something carelessly let fall, sets our nerves tingling, and we give way to sudden expressions of irritability. We have striven successfully against our temper for some time, now a thousand things, too small to name, but not too small to feel, some more than we can possibly bear. This comes in a great measure from the illness itself, and partly that we may have been exerting ourselves too much, and our weakened bodies cannot bear the strain. The fear is at this stage lest we should turn into confirmed grumblers, or express our irritation by a pained look or a fretful tone of voice, which may easily become habitual.

Now we must be very careful not to indulge in these things; they can be resisted to a great extent, and by God's help, can be almost, if not quite, overcome. It is most important for the health of both mind and body not to yield to them, but to cultivate instead a cheerful face and a calm, thankful manner. At the same time we know there are states of suffering which do affect the voice at times and give sharpness to its tone. These are exceptions, but on the general tone of our voices we should keep careful watch, and if anything sounds unnatural, try earnestly to subdue it, by this means we shall soon discover whether it is under our control or not.

Discontent quickly betrays itself in the voice and countenance and manner, and because we are sick we must not think ourselves privileged to indulge in anything wrong, whether in word or deed. It is very desirable to repress all such expressions as, "How long you are," "Do bring that quickly," "I wish you would make haste," for though they often mean nothing, yet they are the straws that show which way the wind blows, and by not indulging in them we are going far to overcome the evil thing itself.

What we should constantly do is to ask our Heavenly Father to set a watch before our mouths and keep the door of our lips; it is the tongue by which we offend, and if we keep that and our hearts with all diligence, we shall be making the right use of a time of trial and conflict to strengthen our faith in God and to show our entire dependence on His unerring love.

### Our Indian Letter.

January 5th, 1892.

Miss M. L. Hayes, who left England on November 26th by P. and O. has been posted to Peshawar; Miss E. R. Green and Miss M. Hammers, who sailed from England by P. and O. on December 3rd, to Quetta; Miss B. L. Cann and Miss M. K. Lawrie are posted to Miau Meer; Miss F. J. Harris, to Peshawar; Miss E. M. Russell and Miss F. C. Walton to Allahabad (these last-named five having embarked from Portsmouth in H.M.S. *Crocodile* on December 11th); and Miss J. Campbell and Miss A. M. Waterhouse have also been appointed, and their destination will be Umbella.

Miss Welchman, late Acting Superintendent, Meerut, who received the medal for the Black Mountain Expedition, and this year received the Red Cross from Her Majesty, is to be married to-morrow, January 6th. Last month, Sister Bates, of Meerut, was also married. Sister Hughes, old nurses of St. Mary's, Paddington, will be sorry to hear, has been suffering from enteric fever, but she is now convalescent.

Last week I read "Another Old Fogie's" epistle, written commenting on mine in your issue of November 21st. I was sorry that she should have so mis-quoted my letter and perverted its meaning. I can pardon her, and make excuses for her; when people get to the "Old Fogey" stage, like her and me, they are liable to these fits of righteous indignation; they are, so to speak, the little enthusiasms of our second youth. I should not like anyone to feel that the Sisters out here are a frivolous set, as "Another Old Fogie" appears to think, from the misconstructions she puts on my letter, so I will try and enlighten her a little on one or two points she seems ignorant of.

Several pairs of gloves are not a very extravagant piece of advice to a lady buying an outfit for five years, and suede and silk are the most economical, kid being destroyed with one wearing on a hot day. Silk gloves will wash when soiled; and even "Another Old Fogie" must admit that gloves are generally considered a necessary adjunct to a lady's outdoor costume. A riding habit is not out of the way. Walking exercise is impossible in most stations, except for three months in the cold season. Outdoor recreation depends on riding or driving. Alas! I am too old and stout to ride, so am dependent on a carriage for my outing. A horse only would be much cheaper. In the hills driving is not allowed. At Simla only two personages are allowed to have carriages, the Viceroy and the Commander-in-Chief; at other hill stations I have been to no one may drive, so "Old Fogeys" who cannot ride, have to be carried about by four coolies in a coffin-like arrangement slung on poles, called a dandy.

I saw one nursing Sister, not of the Indian Nursing Staff, riding in the hills in uniform, and I think a riding habit would have been more decent and becoming. Nor do I think one evening dress that can be adapted to all circumstances too much of a good thing for a gentlewoman's five annual visits to her friends. One more suggestion I would like to make, and that is that she spells "fogey" either *fogy* or *fogey*, as it is more correct than "fogie." I have been through every grade of nursing in civil hospitals at home, from cleaning brasses, polishing lockers, &c., as a probationer, to the superintendence and management of others, and I think even the last paragraph of my letter was not misplaced advice to those recruits who are being sent out from England without superintendence in Her Majesty's troopships or in mail steamboats. "Another Old Fogie" is probably no longer young, but though I am old myself, I have seen the temptations that young ladies are put to in nearly a whole month's total idleness, without any work to do, and human nature is the same all the world over, and needs guiding, whether under the soldier's coat or the nurse's cloak.

As regards the social rules of the Indian Nursing Staff, they are very strict. When a Sister is attached to a station for duty she is rarely permitted to go to a dance, as "late hours and dancing" are not considered suitable for nursing Sisters. They never go out into society alone, the custom being for two to go together, and when not possible for two, they usually, if not always, have the chaperonage of a married lady.

I hope to send a more interesting letter next time, but I am tired this week; my wards have been very heavy. Enteric fever is bad again, and I have had little rest. Last year, at my present station, on Christmas Day, the hospital was the scene of disgraceful uproars and drunkenness. There were no "Sisters" here in those days. This year a guard was placed round each hospital block to prevent liquor being brought in or patients getting out; it is no easy matter to secure discipline in a hospital in three separate blocks, 500 yards apart from each other, and the only hospital officers on duty for the day (except for about three hours) being the Sister and the Apothecary. I came on duty soon after six a.m., and left the hospital at midnight, having a few hours' rest for mealtimes. A lady's restraining presence had some effect; only one patient was known to be drunk, and all said what a quiet Christmas it had been. A large number of the patients I entertained at tea in one of the wards, which was decorated with bunting for the occasion, the patients' principal pleasure being the delight of having real English damask table-cloths, being allowed to sugar and milk their own tea, and drinking out of cups and saucers. Those in bed had my best linen damask napkins on the bedside tables. One Tommy exclaimed, "Oh! ain't it just like 'ome, the rattle o' the cups and saucers." One poor Tommy thought so much about the real home-made tea, and the preparations generally, that he got tired and fell asleep before it came off, and was too sleepy to eat anything.

The Tommies here do appreciate having a woman about them when they are ill; they are now looking forward to other Sisters coming to the station. One poor consumptive patient implored me to stay, to try and keep them from being "too bobbery" to them, or "too regimental." They are very like children—manage them the right way and you can make them do anything. They now keep their beds tidy, they have given over spitting all over the floor, as they did when first I came; contraband goods have been banished from the wards; coarse language stopped—not from any fear of me, but from respect on their part. An old patient will say, "Don't do that, Pat; our Sister does not like it." One poor dying man yesterday drank something I gave him after refusing all food from the orderly, and said, "I drink it only to please you, Sister." He died a few hours after.

### Be not Weary.

Tho' toil be often dreary,
And patience well-nigh gone,
Still let us not be weary,
But struggle bravely on.
In due time, if we faint not,
We shall reap a full reward;
And, oh! the joy and pleasure
Of working for our Lord!

Christ, be Thou ever near us,
To help us in our strife.
To Thy sweet Presence bear us
When we end our earthly life;
That, all our troubles over,
We may worship and adore,
And with Thy holy angels,
Serve Thee for ever more.

Sister Kate.

### Everybody's Opinion.

[*Correspondence on all subjects is invited, but we cannot in any way be responsible for the opinions expressed by our correspondents. No communications can be entertained if the name and address of the correspondent is not given, or unless one side of the paper only be written on.*]

### NURSING AT WEST COWES.

"THE TREASURER" writes: As it was through the medium of your very valuable paper we succeeded in getting a district nurse for the poor of West Cowes, will you kindly insert the following news therein: Mrs. de Horsey, of Melcombe House, West Cowes, started the idea of having a thoroughly trained nurse to nurse amongst the poor, knowing from what she saw how much such was needed, and what great suffering the poor bed-ridden folks and others had to bear, who could not afford a nurse, yet would be so extremely ill, and oftentimes valuable lives in danger amongst the working men's homes; and sad to say, deaths occurring which kind and careful nursing and nourishment would save. Mrs. de Horsey, called a drawing-room meeting amongst some ladies, when the question was quietly discussed, and each one heartily entered into the plan, all taking some part, but the thanks are due to the promoter of the scheme for the funds flowing in so freely from the many kind-hearted ladies and gentlemen who yearly visit this charming watering place, for their kind support and liberal help so readily accorded to that lady. Our most kind Queen was graciously pleased to say she was much interested in the matter, and contributed the sum of five pounds towards the funds for providing a nurse and nourishment to the poor suffering ones, and there are many. Indeed the scheme has proved so successful that the nurse, who has already made herself liked, is the Sister Francis Cole, who was nursing Sister at Rickmansworth and Warefield for eight years, winning laurels there amongst all classes; and also formerly Matron of the Cottage Hospital, Dartmouth. I may say the good work was begun on December 1st, 1891, and is proving a great benefit.

### AN APPEAL TO THE BENEVOLENT.

A FULLY-TRAINED hospital monthly nurse of eleven years' proved usefulness is now suffering from an internal complaint, which has taken all her savings. She is at present in the workhouse, and has neither relations nor means. Only thirty-six years old, and she can never return to her former employment. A fellow nurse is anxious to give her a home, but has not the means to support her. She can furnish full particulars to all who are willing to give their help in providing a home as soon as possible. "Bis dat qui cito dat." Address Harriet Middleton, Moor House, Womersley Road, Crouch Hill, N.

[We have enquired into the above case and seen the certificate and testimonials of the nurse described.—ED.]

### BRENTFORD DISTRICT SCHOOLS.

GEORGE WILLIAMS, writes: In your issue of the 16th inst. under the heading "Real Scandals" it is stated that "The nurse of the Brentford Union stated to the guardians on January 6th that she was so overworked she had not been to bed since December 20th." As I have been subjected to much annoyance by persons assuming that these remarks referred to the nurse at these schools, may I ask you to be good enough to deny in your next issue that anything of the kind has occurred in this institution?

### Death in our Ranks.

WE regret to announce the death of Nurse Emily Horne, Probationer at the Cumberland Infirmary, Carlisle, on February 1st, from pneumonia after influenza.

### Presentations.

CITY HOSPITAL, EDINBURGH.—On the occasion of her birthday the servants of the institution presented Miss Mackay, the Matron, with a handsome reading lamp, an ivory set of hair brushes, and an ivory hand mirror as a token of the high esteem in which she is held by them.

THE prizes left to the Royal College of Physicians by the late Sir Alexander Morison, a former President, to be given to one male and one female attendant for meritorious attendance on the insane, have this year been awarded by the Council, on the recommendation of Dr. Batty Tuke, the Morison lecturer for 1891, to Robert Emslie, head attendant, Montrose Royal Asylum, for long and faithful service; and to Helen Matthew, head nurse, Saughton Hall Institution for the Cure and Care of the Insane, generally for zealous and faithful service, and specially for having gone through a systematic course of training as a nurse in a general hospital and having gained a certificate.

### Appointment.

[It is requested that successful candidates will send a copy of their applications and testimonials, with date of election, to THE EDITOR, The Lodge, Porchester Square, W.]

LONDON FEVER HOSPITAL.—Miss E. A. Morgan has been appointed Night Superintendent of this hospital. Miss Morgan was trained at the Royal Infirmary, Edinburgh, and has been Sister at St. Marylebone Infirmary, Notting Hill, for more than a year. We congratulate Miss Morgan on her appointment, and wish her every success.

### "Sister is Wanted."

"SISTER is wanted!" Swift as thought she came,
Help in her hands and comfort in her face,
Down the white ward lit by the lamp's pale flame,
To where *It* lay, a sore and sad disgrace
To proud humanity. From the street's foulest place
Sin, want, and sorrow, all too clearly told,
In the thin form and garments' matted fold.
Swift to the bedside to her aid there comes
Science, skill, wealth—the outcast of the slums
Shall not die now if highest skill find grace.
"Sister is wanted"! By the door, ah see!
The Queen's physician; softly, "Come," says he,
"Now to the royal palace; watch you there,
Beside this bed, 'Sister is wanted here.'"

### Notes and Queries.

TO CORRESPONDENTS.—1. Questions or answers may be written on post-cards. 2. Advertisements in disguise are inadmissible. 3. In answering a query please quote the number. 4. A private answer can only be sent in urgent cases, and then a stamped addressed envelope must be enclosed. 5. Every communication must be accompanied by the writer's full name and address, not necessarily for publication. 6. Correspondents are requested to help their fellow nurses by answering such queries as they can.

### Query.

*Home Wanted.*—Can anyone tell me of a home where a little girl, blind, crippled, and subject to fits, could be received?—*Sister Katherine, Nurses' Home, Plaistow, E.*

### Answers.

*Ottershaw Fever Hospital.*—We have never mentioned this institution in our pages. As you do not give name, address, or date we cannot publish your communication.

*Examination Questions.*—So many answers having been received to the last question, we cannot award the prize till next week.

*C. McR.*—2s. received with thanks.

*H.M.'s Nursing Sisters.*—Government allows £15 for outfit for a Sister going to India; this is ample to cover all *uniform* requisites. The amount of uniform needed varies according to the station; in England two frocks a-year are enough, and half-a-dozen caps and a dozen aprons will last two years.—*Kate.*

### The Blind Man's Dog.

"My dear Mary, why do you let that dog pull at your skirt? He will tear it! I have noticed at every street crossing he has done that."

The speaker was an elderly, thin lady with eyeglasses. Her dress was more stylish than neat, and her manner was that of an altogether superior person. Her companion whom she addressed was young, and possessed of a pleasing figure and winning face; the most noticeable thing about her being a quiet air of reserve and refinement. She was dressed simply in black.

"My dear Aunt Rhoda," she replied, "my dress is old, and I would not mind much if it should get torn, but Peter—eh, Peter?—Peter has never torn a skirt of mine in his life, and he always does this."

"You're a great deal too good-natured, Mary—it's your fault! However, it's well for you to have plenty of money to buy new dresses with when you want them. Now, my girls——"

"Oh, but they don't like dogs, I know."

"You are right there. I have always taught them that the taste for dogs is a queer, unnatural taste, and not to be encouraged. Why, just think of the inconvenience of having a dog in the house! the amount of time wasted in taking it for walks, feeding it, cleaning it, cleaning up after it, perfuming the house to sweeten the doggy smell—all that trouble and fuss for the sake of a stupid animal whose one idea in life is food, and who has no more sense than—than——"

"Than some human beings, Auntie?"

"My dear, you are too ready to make fun of people. The idea of comparing any human being to a soulless dog!"

"Aunt, if I were to tell you the story of Peter here, and how and why he learnt that habit of pulling people across streets, you wouldn't account him soulless."

"Break him of it, Mary; it's a bad habit. Dear me, I wonder have I dropped that!"

"What?"

"The pencil-case you gave me to take to Eva. Oh, no; here it is. That wretched dog has made me quite stupid."

"Now, Auntie, I'd like to tell you about Peter."

"If you like, dear—go on. Oh! look at those lovely feathers. That pale green one is just what Blanche wants for her bonnet. I wish I could afford to buy it for her."

"Come along into the shop, Auntie. I'll make her a present of it."

"Oh, no; I couldn't think of letting you——."

"Come along."

Mary took her aunt's arm, and aunt, niece, and Peter disappeared into the shop for a few minutes. When they emerged again the aunt was speaking—

"Of course, it was just as well for me to carry it, it is so light. Blanche can sew it in her hat this afternoon."

"Well, now, Auntie," Mary said, "I'm going to tell you about Peter. It was twelve months ago—before mother died. She and I were out walking, when we saw a blind man—let us turn up here, Westbourne Grove is so crowded. Well, this blind man held a stick with which he felt about htly, but he seemed to place much more reliance on a little dog, which he held by a string. Eh, Peter? The poor man was very old, and evidently poverty-stricken, and mother and I stayed to watch him. We were interested, besides, in the dog. Do you know that dog led his master through the most crowded parts of the street just as well as any human being, and when he came to a crossing that dog stopped, and his master, feeling the string loosened, stopped also, until at last, when the carriages and carts had passed and the way was clear, that soulless dog, as you would call him, gave a great tug at his string, as much as to say, 'Come along,' and pulled across the street as fast as ever the poor old man's tottering legs could follow. Peter was that dog. He wants to lead me across the street in the same way, and if there's any danger, he pulls me back instead of forward—dear old Peter!"

"Dear me!" the aunt exclaimed. "I've often heard these stories, but I never believed them. I suppose you bought him from the old beggar?"

"What! Take away the poor old man's crutch—his eyes? No, indeed. It was very sad. I made enquiries the very next day, and went to where the man lived. Alas! he had died during the night. But I buried him and brought away his dog to take care of. And now, Aunt, can you imagine the depth of confidence that blind man must have placed in his dog! Think of your Blanche now, how timid she is about crossing a street. Even if her own brother has her tightly by the arm, still she is frightened. Imagine her—anybody—being blind, hearing the sound of cart-wheels and horses' hoofs on every side, and yet trusting implicitly to the intelligence of a little dog to guide them, like that old man."

"It does seem wonderful, certainly."

"I thought I should alter your opinion about dogs, Aunt; but I must leave you now, and hurry home. Some people are coming to see me."

The elderly lady watched her niece disappearing, with Peter at her heels, and then betook her own homeward way, looking not particularly amiable. Two of her daughters opened the door to her.

"Here, Blanche, here's a feather I got Mary to buy you when I was out, and I also persuaded her to buy you a pencil-case, Eva," she said to them. "Where's Rose?"

"Oh, she's gone out; she'll be in again in a minute," one of the girls answered. "How's Mary?"

"Oh, very well, dear; always pottering about with beggars. She was telling me about a blind man she patronized; he died, and that dog, Peter, was his."

"She never told us," the girls cried.

"No," their mother said, "she was too much taken up with her beggars. She never seems to think that charity begins at home. I think she might remember you more. Oh, there's Rose. Well, Rose, where have you been?"

"I looked in at Mary's on my way home. She is giving a dinner, or something, to a lot of poor people—crowds of them."

"There you are, my dear daughters; that's just what I was saying. Mary's all for the beggars, and forgets her own. Why, what's this?"

A servant entered the room with a large hamper. "With Miss Mary's compliments, ma'am," she said. The girls sprang eagerly to unfasten the basket, and scattered its contents out upon the table. Their exclamations of delight were many as they counted the good things.

"Oh, of course," their mother said. "I remember now. I was telling Mary about our little dinner party to-morrow night, and I suppose she has sent this in consequence."

"Any answer, ma'am?" the servant asked.

"No—yes, many thanks!"

## Figures and Tables

**Figure f1:**